# Effects of red blood cell transfusion on cerebral hemodynamics of preterm neonates

**DOI:** 10.1117/1.NPh.11.4.045014

**Published:** 2024-12-17

**Authors:** Caterina Amendola, Agnese De Carli, Tiziana Boggini, Davide Contini, Sofia Passera, Nicola Pesenti, Lorenzo Spinelli, Martina Giovannella, Turgut Durduran, Udo M. Weigel, Alessandro Torricelli, Gorm Greisen, Monica Fumagalli

**Affiliations:** a Politecnico di Milano, Dipartimento di Fisica, Milan, Italy; b NICU Fondazione IRCCS Ca’ Granda Ospedale Maggiore Policlinico Milan, Milan, Italy; c University of Milano-Bicocca, Department of Statistics and Quantitative Methods, Division of Biostatistics, Epidemiology and Public Health, Milan, Italy; d Istituto di Fotonica e Nanotecnologie, Consiglio Nazionale delle Ricerche, Milan, Italy; e ICFO – Institut de Ciències Fotòniques, The Barcelona Institute of Science and Technology, Casteldefells, Spain; f ICREA – Institució Catalana de Recerca i Estudis Avançats, Barcelona, Spain; g HemoPhotonics SL, Casteldefells, Spain; h Rigshospitalet and University of Copenhagen, Department of Neonatology, Copenhagen, Denmark; i University of Milan, Department of Clinical Sciences and Community Health, Milan, Italy

**Keywords:** preterm neonates, NIRS, time domain NIRS, diffuse correlation spectroscopy, cerebral hemodynamics, cerebral metabolic rate of oxygen extraction

## Abstract

**Significance:**

Anemia is a common problem in preterm neonates, and red blood cell transfusion (RBCT) is used to improve oxygen delivery. However, RBCT is associated with complications, although an increase in cerebral oxygenation has been documented, and no universally accepted biomarker for the need for transfusion (i.e., the concentration of hemoglobin in the blood) has been defined.

**Aim:**

We used a hybrid optical device (BabyLux device) that merges time-domain near-infrared spectroscopy (TD-NIRS) and diffuse correlation spectroscopy (DCS) to potentially obtain a better assessment of the cerebral effects of RBCT compared with previous studies using continuous wave (CW) spatially resolved NIRS.

**Approach:**

Eighteen clinically stable preterm neonates were assessed before and after RBCT by the BabyLux device as five repetitions of 60 s measurement (with 1 s acquisition time), estimating the cerebral blood flow (CBF) as a blood flow index (BFI), the total hemoglobin concentration (tHb), and the cerebral tissue oxygen saturation ($S_{t}O_{2}$). $S_{t}O_{2}$ was also continuously monitored by a commercial CW-NIRS device, as well as peripheral saturation, $S_{p}O_{2}$. Tissue oxygen extraction (TOE) and cerebral metabolic rate of oxygen consumption (${\mathrm{tCMRO}}_{2}$) were computed, and the Wilcoxon signed-rank test for paired data was performed, comparing the data acquired before and after RBCT.

**Results:**

The BabyLux data from four neonates did not meet quality criteria and were discarded. After the transfusion, tHb and $S_{t}O_{2}$ (measured both with TD-NIRS and CW-NIRS devices) significantly increased, causing a significant decrease in TOE. CW-NIRS showed a wider dispersion of $S_{t}O_{2}$ data compared with TD-NIRS. However, CBF did not decrease proportionally but the variation was high, as well as for ${\mathrm{tCMRO}}_{2}$.

**Conclusions:**

The results confirm previous CW-NIRS studies, but the wide variability of BFI makes the effects of RBCT on cerebral metabolism uncertain.

## 1 Introduction

Anemia is a common problem in preterm infants and up to 90% of extremely preterm neonates (<28 weeks’ gestation) receive at least one red blood cells transfusion (RBCT) during their hospitalization in the neonatal intensive care unit (NICU).^1^ It is referred to as “anemia of prematurity” and is mainly caused by insufficient RBC production during growth and by RBC losses both spontaneously and iatrogenically (blood samples for clinical care).

Anemia can lead to impaired oxygen supply to organs with the main concern for the developing brain, due to its vulnerability related to the high rate of growth and cellular development. In clinical practice, RBCT improves weight gain, reduces supplemental oxygen need, and intermittent apnea and bradycardia. RBCT, however, has complications.^2^^–^^7^

Although the benefits and risks of RBCT have been widely described, the optimal threshold for RCBT need is still debated; usually, transfusion guidelines recommend hemoglobin (Hb) and hematocrit (Hct) thresholds according to age and need for respiratory support.

Two recent trials and a meta-analysis addressed the issue of the threshold of transfusion comparing a “liberal” versus a “restrictive” policy and showed similar results in terms of survival and neurocognitive outcome.^8^^–^^10^ This evidence suggests that the Hb level in the blood measured by hematocrit may not be the best clinical biomarker for RBCT needs. Two studies did not find a correlation between Hb or Hct and cerebral or peripheral oxygenation,^11^^,^^12^ whereas Van Hoften did so before the transfusion; however, they did not identify a threshold below which tissue perfusion and oxygenation are compromised.^13^

Other biomarkers have been studied for their suitability as “need for RBCT” discriminators:

• Serum lactate: it is one of the final products of the anaerobic metabolism of cells; thus, it is elevated in all conditions of low tissue perfusion or oxygenation. Several studies have been conducted to define serum lactate as the biochemical trigger for RBCT in neonates.^14^^–^^19^ However, none of these studies reported a significant correlation between Hb before the transfusion and serum lactate, although this does not rule out that serum lactate may be more relevant than Hb.
• RBC volume: this is the total volume of RBC in all circulating blood. A positive correlation has been found between RBC volume and cardiac output of anemic neonates.^20^ Indeed, Hb and Hct may present normal values with very low RBC volume (when total blood volume is low).^21^ However, assessing RBC volume is time-consuming, it requires blood transfusion or the use of a contrast agent, and it is not feasible in preterm neonates.
• Doppler ultrasound: blood flow and velocity in vessels are dependent on many parameters, such as cardiac contractility, diameter of the vessels, and viscosity of the blood, and parameters to define the need for RBCT have not been identified.^14^^,^^22^^–^^24^

Since 1999, near-infrared spectroscopy (NIRS) has been used to study the effects of RBCT: Wardle et al.^12^ used Hamamatsu NIRO 500 (Hamamatsu K.K. Ltd., Hamamatsu, Japan) and a pulse oximeter (Datex, Finland) to study the tissue oxygen extraction (TOE) and metabolism in 15 preterm neonates that suffered hypotension, before and after RBCT treatment. Among these 15 neonates, two were treated with RBCT. They measured blood flow as well as venous oxygen saturation in the forearm by venous and arterial occlusions. After this study, several works have been published on the effects of RBCT on the hemodynamics of preterm infants (the review of Kalteren et al. gives a detailed description^25^), and they demonstrated that RBC transfusions cause an increase in tissue oxygen saturation ($S_{t}O_{2}$)—in particular, in the brain (cerebral oxygenation). However, most of the studies failed to determine a threshold that could be useful to define the need for an RBCT. This was mostly due to the wide range of values of measured TOE and $S_{t}O_{2}$.

Recently, cerebral and mesenteric NIRS data^26^ from a subset of infants enrolled in the TOP (Transfusion Of Prematurity) trial^8^ revealed that cerebral and mesenteric saturation increases and TOE decreases after a transfusion, with a greater difference for the lower Hb threshold group; pre-transfusion cerebral saturation below 50% was associated to adverse outcome (death/NDI at 22 to 26 months corrected age).

Up to now, most studies were performed using continuous wave NIRS (CW-NIRS) devices, typically adopting the space-resolved spectroscopy (SRS) approach. SRS NIRS devices can give an estimate of cerebral oxygen saturation if a calibration factor (the differential pathlength factor, DPF) is used to rule out the effect of light scattering on photon pathlength in the tissue. The DPF is typically measured in a few subjects by using frequency domain (FD) or time domain (TD) NIRS and literature data are used instead of individual (subject-specific) data. The use of TD NIRS can provide an estimate of Hb and $S_{t}O_{2}$ without the need for such calibration. In addition, by combining TD-NIRS^27^ with diffuse correlation spectroscopy (DCS),^28^ it is possible to simultaneously measure $S_{t}O_{2}$ and a blood flow index (BFI)—a surrogate of CBF—and thereby estimate the cerebral metabolic rate of oxygen consumption (${\mathrm{tCMRO}}_{2}$). This is relevant because when oxygen delivery to tissue becomes insufficient, ${\mathrm{tCMRO}}_{2}$ will fall. So, ${\mathrm{tCMRO}}_{2}$ is a potential biomarker.

The aim of this study was to assess the effects of RBCT on brain hemodynamics in preterm neonates by means of a hybrid TD-NIRS and DCS device: the BabyLux device.^29^^,^^30^ As monitoring over time is not practical, due to the design of the sensor, a commercially available SRS CW-NIRS device (INVOS 5100C, Somanetics Corporation, Troy, Michigan, United States) was also used, and a secondary aim was to compare the devices.

## 2 Materials and Methods

Measurements were performed at Fondazione IRCCS Ca’ Granda, Ospedale Maggiore Policlinico (Milan, Italy) from 2019 to 2022.

### 2.1 Study Protocol

The protocol was approved by the Comitato Etico Milano Area 2 and registered at ClinicalTrials.gov, identifier NCT03983694. Written informed parental consent was obtained before the infant’s enrolment.

The inclusion criteria were RBCT prescribed according to local guidelines and corrected age <4 weeks.

No exclusion criteria were defined.

Cerebral oxygenation was recorded with a commercially available SRS CW-NIRS device (INVOS 5100C, Covidien Inc., Minneapolis, United States). The device operates at 730 and 830 nm, with an integration time of 1 s, and it provides two source-detection separations: 3 and 4 cm. The sensor was placed on the prefrontal region of the neonate’s head and not moved during the study. The before and after measurements were averages of ∼30  min immediately before the start and after the end of the transfusion. Depending on the total volume of transfused blood and the weight of each neonate, the RBCT itself lasted at least 4 h. However, the CW-NIRS and BabyLux measurements were not conducted simultaneously. The CW-NIRS recording was paused for the entire duration of BabyLux measurements to prevent crosstalk between the two devices, which could lead to a non-negligible increase in the TD-NIRS background signal.

TD-NIRS and DCS measurements were performed with the BabyLux device to assess cerebral oxygenation and perfusion before and after RBCTs. The device operates at 690, 760, and 830 nm, with an integration time of 1 s. Measurements, consisting of five 60-s repetitions, were conducted both before and after the transfusion, with a maximum of 30 min between the measurements and the transfusion. A source-detection separation of 1.5 cm was used. The probe was placed in the same location on the fronto-parietal region of the head, but opposite side of the INVOS probe. During the measurements, the BabyLux probe was fixed with a black elastic bandage. To avoid direct light to affect measurements and to further reduce risks (of eye damage), a capacitive sensor was used to immediately stop the laser emission in case of no-contact with the skin is detected. Moreover, to assure the sterilization, a sterile ultrasound probe cover was used during measurement.

Heart rate (HR) and arterial saturation ($S_{p}O_{2}$) were continuously monitored according to clinical practice using Radical-7 Pulse CO-Oxymeter (Masimo, Irvine, CA, United States) or Philips Intellivue X2 (Philips, Amsterdam, The Netherlands) and continuously recorded. Moreover, invasive blood pressure was recorded if available; otherwise, non-invasive blood pressure measurements were performed three times before and after the transfusion.

Synchronization of peripheral saturation and BabyLux data was performed retrospectively by aligning the acquisition times from each device.

Capillary hemoglobin concentration was measured before and after the transfusion. The blood samples were within 6 h before the transfusion, and 1 h after the transfusion.

### 2.2 Data Analysis

TD-NIRS data were analyzed using the semi-infinite homogeneous model for photon migration,^31^^,^^32^ and the optical properties (absorption coefficient and reduced scattering coefficient) at the three wavelengths were retrieved. From the wavelength-dependent absorption coefficients, by means of Beer’s law, and assuming a water content of 90%, $O_{2}\mathrm{Hb},\mathrm{HHb}$, $\mathrm{tHb}=O_{2}\mathrm{Hb}+\mathrm{HHb}$, and $S_{t}O_{2}=O_{2}\mathrm{Hb}/\mathrm{tHb}$ were calculated.^33^

Concerning BFI, the DCS data were analyzed using the semi-infinite homogeneous model for the electric field autocorrelation function (retrieved from the intensity autocorrelation function through the Siegert relation).^34^^,^^35^ The optical properties used to retrieve the BFI were the ones measured with the TD-NIRS module at 760 nm and averaged among the measurements performed before and after the transfusion on the same neonate. However, BFI measured by DCS is expressed in a non-traditional unit ${\mathrm{cm}}^{2}/s$; thus, to convert it into a more conventional unit for cerebral blood flow (CBF, ml/100g/min), the BFI measured by DCS was calibrated with the coefficient estimated by Giovannella et al. in newborn piglets.^36^ The signal quality exclusion criteria are the ones reported in our previous work, where similar data analysis was performed.^37^

These results were combined with the concentration of hemoglobin in the blood [Hb], and with $S_{p}O_{2}$, to obtain $\mathrm{TOE}=(S_{p}O_{2}-S_{t}O_{2})$ and the index of the cerebral metabolic rate of oxygen, ${\mathrm{tCMRO}}_{2}=\mathrm{TOE}\cdot \mathrm{CBF}\cdot k\cdot [\mathrm{Hb}]$, where k is the oxygen-carrying capacity of hemoglobin (k=1.39  ml of $O_{2}$ per g of Hb).

To compute TOE and ${\mathrm{tCMRO}}_{2}$, all signals were synchronized (after recording), and the ${\mathrm{tCMRO}}_{2}$ was estimated for each second of measurement, overall results (optical and hemodynamic parameters) for “before” and “after” transfusion were obtained as the grand mean of measurement during the five repetitions of 1-min recordings by the BabyLux device. The results obtained before and after the transfusions were compared with the paired Wilkinson signed-rank test.

## 3 Results

Eighteen preterm neonates were enrolled in the study; the enrollment was stopped before reaching the calculated sample size (N=30) due to a fault in an electronic component of the BabyLux device, which was not possible to replace in a reasonable time due to global shortage in electronic components.

Four of the eighteen recruited neonates were excluded according to BabyLux signal quality exclusion criteria: a low number of photons collected ($N<10^{4}$), to guarantee proper signal-to-noise ratio; low quality of fitting parameter: $\chi^{2}<0.5$ and $\chi^{2}>5$, to reinforce the validity of the retrieved results; small values of reduced scattering coefficients: $\mu_{s}^{\prime }<3\;{\mathrm{cm}}^{-1}$, to avoid data affected by direct light, i.e., light that does not penetrate neonates’ tissues but is directly collected by detection fiber. There was no capillary sample for the three babies, and we decided to use venous hemoglobin. For one baby, the blood sample after RBCT was performed the day after the procedure.

The 14 babies were typical for anemia of prematurity, and the hemoglobin rise (from 8.3 to 11.8  g/dl) was as expected (Table 1).

**Table 1:** Information on patients enrolled in the clinical study (N=14).

GA at birth (weeks), mean (SD)	30.8 (3.1)
GA at enrollment (weeks), mean (SD)	35.6 (3.1)
Weight at birth (g), mean (SD)	1250 (640)
Weight at enrollment (g), mean (SD)	1801 (595)
Male, n (%)	7 (50)
Hb before transfusion (g/dl), mean (SD)	8.3 (0.8)
Hb after transfusion (g/dl), mean (SD)	11.8 (1.2)
Number of RBCT before enrollment, mean (SD)	2.7 (2.3)

**Fig. 1:**
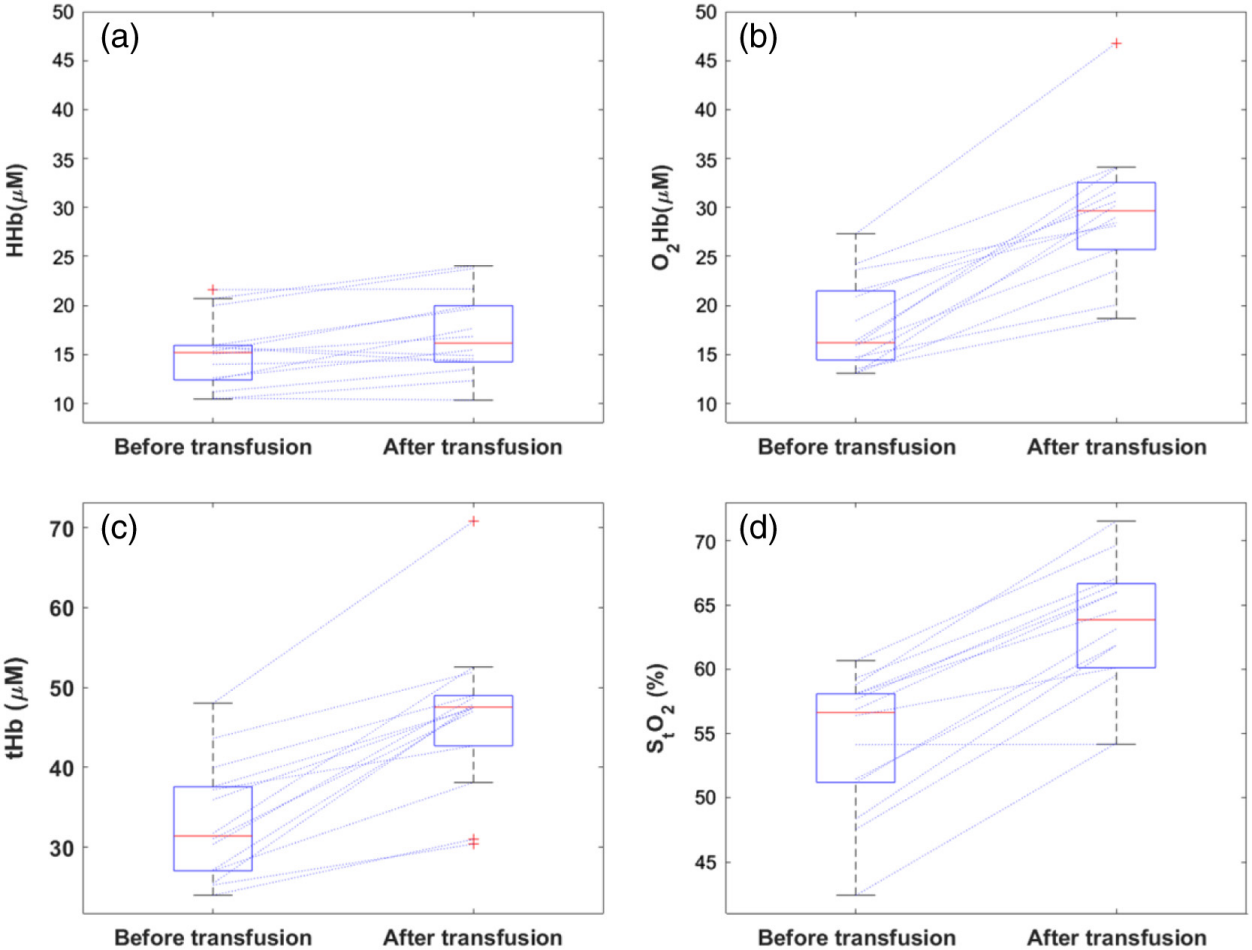
Box plot of HHb (a), $O_{2}\mathrm{Hb}$ (b), tHb (c), and $S_{t}O_{2}$ (d,) measured with TD-NIRS, before and after RBCT. The red lines inside the box represent the median, and the bottom and top edges of the box indicate the 25th and 75th percentiles, respectively. The whiskers extend to the most extreme data points not considered outliers, and the outliers are plotted individually in red. Blue lines connect values measured before and after the transfusion for the same patient.

Cerebral oxygenation as measured by the TD-NIRS increased after RBCT (Fig. 1). The median HHb and $O_{2}\mathrm{Hb}$ increased from 15.2 to 16.2  μM (+17.1%), and 16.2 to 29.7  μM (+ 66.1%), respectively. The median $S_{t}O_{2}$ from 56.6% to 63.9% for (+15.8%). The median tHb also increased from 31.4 to 47.5  μM for (+36.5%) (Table 2).

**Table 2:** Median values of hemodynamic parameters, their relative variation before and after the transfusion, their interquartile ranges, and the p value obtained with the Wilcoxon signed-rank test are reported.

	Before transfusion	After transfusion	Relative change (%)	P value
HHb (μM)	15.2 (12.5; 15.9)	16.2 (14.3; 19.9)	+17.1 (1.2; 22.2)	<0.01
$O_{2}\mathrm{Hb}$ (μM)	16.2 (14.5; 19.9)	29.7 (26.3; 32.3)	+66.1 (38.6; 93.9)	<0.01
tHb (μM)	31.4 (27.1;37.5)	47.5 (43.8; 48.9)	+36.5 (23.5; 58.2)	<0.01
$S_{t}O_{2}$ TD-NIRS (%)	56.6 (51.3; 58.1)	63.9 (60.6; 66.5)	+15.8 (13.2; 22.9)	<0.01
$S_{t}O_{2}$ CW-NIRS (%)	61.9 (54.0; 65.7)	67.9 (62.4; 74.1)	+10.6 (7.9; 18.2)	<0.01
$S_{p}O_{2}$ (%)	94.9 (94.0; 96.7)	96.3 (94.2; 97.9)	+0.6 (−1.8; 4.1)	0.29
TOE (%)	41.4 (35.0; 45.8)	31.0 (28.9; 36.2)	−22.3 (−26.3; −9.5)	<0.01
CBF (ml/100g/min)	37.7 (35.2; 39.6)	34.2 (22.2; 40.1)	−13.5 (−17.0; −1.4)	0.11
${\mathrm{tCMRO}}_{2}$ (ml/100g/min)	1.7 (1.6; 2.1)	1.8 (1.1; 2.0)	+7.1 (−6.98; 11.48)	0.54

**Fig. 2:**
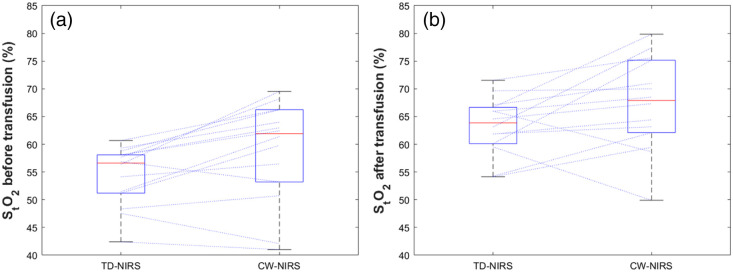
Box plot of $S_{t}O_{2}$ measured with TD-NIRS and CW-NIRS device before the transfusion (a) and after the transfusion (b). The red lines inside the box represent the median, the bottom and top edges of the box indicate the 25th and 75th percentiles, respectively. The whiskers extend to the most extreme data points not considered outliers, and the outliers are plotted individually in red. Blue lines connect values measured with the two devices for the same patient.

Cerebral oxygenation estimates were lower by TD-NIRS than by CW-NIRS, before as well as after RBCT, and had less inter-individual variability (Fig. 2 and Table 2). A Wilcoxon signed-rank test was performed, demonstrating a significant statistical difference between data measured with the two devices (p<0.01), and a significant (p<0.01) correlation was observed with Spearman’s correlation coefficient $R^{2}=0.73$. In summary, the statistical independence of the Wilcoxon signed-rank test, and the strong positive Spearman’s correlation, suggests that the two variables are significantly different, even if they are correlated: although the variables change together, TD-NIRS data consistently has lower values than the CW-NIRS data. The percentage increase of $S_{t}O_{2}$ after RBCT, as measured by TD-NIRS (+15.8%) was higher compared with that measured by CW-NIRS (+10.6%) and had a smaller variation, but this difference was not statistically significant (p=0.2).

**Fig. 3:**
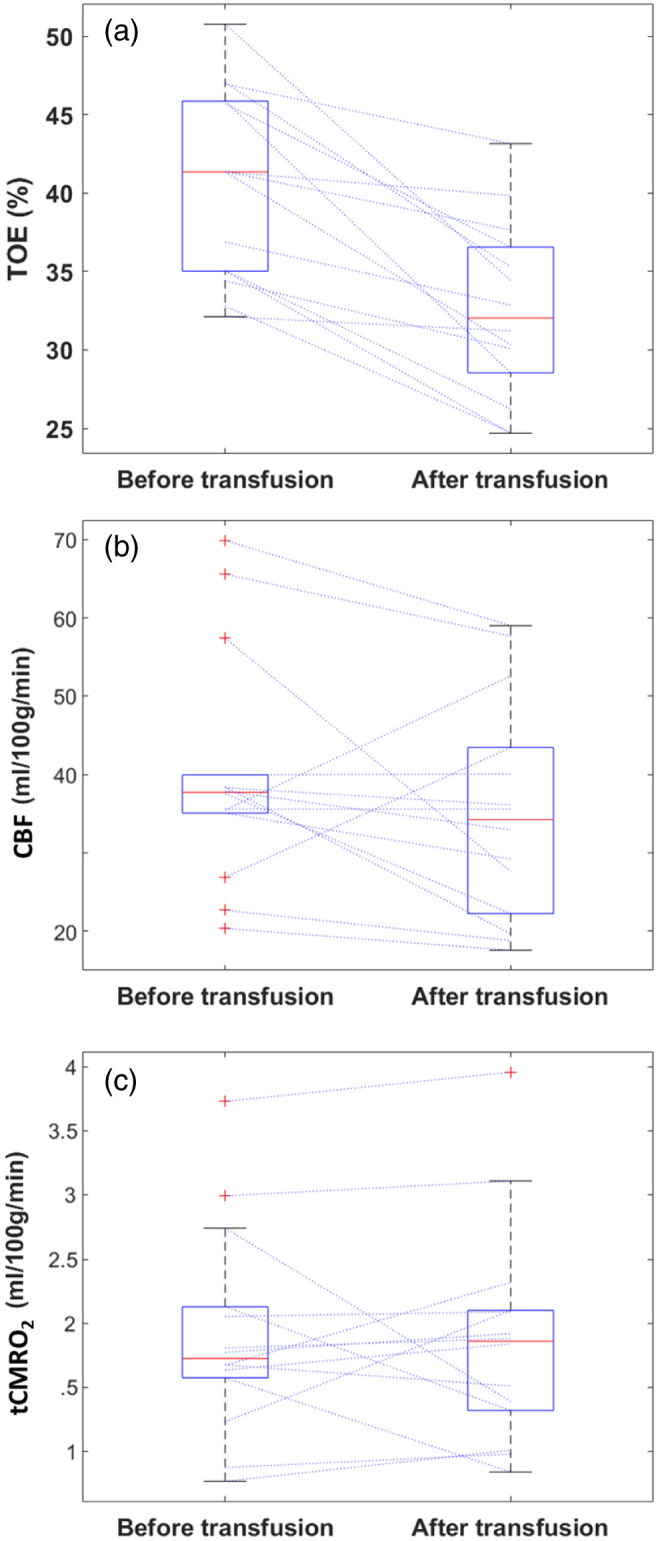
Box plot of TOE (a), CBF (b), and tCMRO2 (c) measured with the BabyLux device before and after RBCT. The red lines inside the box represent the median, the bottom and top edges of the box indicate the 25th and 75th percentiles, respectively. The whiskers extend to the most extreme data points not considered outliers, and the outliers are plotted individually in red. Dashed lines connect values measured before and after the transfusion for the same patient.

After the transfusion, TOE fell from 41.4% to 31.0% (−22.3%) (Fig. 3 and Table 2). By contrast, CBF and ${\mathrm{tCMRO}}_{2}$ did not change significantly. Neither did $S_{p}O_{2}$, HR, or MABP (data not reported).

## 4 Discussion

The TD NIRS results confirm that cerebral $S_{t}O_{2}$ increases after RBCT, at least in stable preterm infants. Surprisingly, the DCS estimate of perfusion did not decrease significantly. Disappointingly, the variability of the measure of BFI and consequently of ${\mathrm{tCMRO}}_{2}$, inter- and intra-subjects, was so large that any meaningful interpretation in terms of oxygen sufficiency before transfusion is impossible, suggesting that further investigation on cerebral hemodynamics during RBCTs might be necessary.

### 4.1 Strengths and Limitations

The sample size is limited. Although the confirmation of the increase in cerebral oxygenation can be considered firm, the results as regards the effects on the blood flow and metabolic rate had very wide confidence limits. The sample size was too small to consider subgroup analysis or any other exploring of the inter-individual differences in the response to transfusion, so the aim of the study to help define a better indication for RBCT in well, growing preterm infants was not reached.

Due to the current construction of the TD-NIRS and DCS optodes (with light fibers), it is not possible to do effective long-term monitoring and studying effects of interventions over hours or days, and therefore, the estimate of the effect of transfusion includes the error introduced by removal/replacement of the probe. By contrast, the CW-NIRS sensor could stay on for the entire study period; however, the variability in responses to transfusion appeared higher, rather than lower, than by TD-NIRS, suggesting that some time-dependent, unexplained variation was present in the CW-NIRS signal.

The re-siting imprecision of DCS, unfortunately, was much higher than that of TD-NIRS in the term newborn study, as already established by Giovannella et al.,^28^ and the data in this study confirm that this is a problem. A previous study performed on anesthetized and paralyzed piglets^35^ showed a lower error in probe repositioning; thus, one likely reason is the movement of healthy infants in clinical studies.

For the purpose of the present study, the problem of the probe replacement variability could have been reduced by not removing the BabyLux sensor during the study, measuring cerebral hemodynamic variations also during the transfusion. However, for the purpose of clinical use, using cerebral oximetry and CBF to judge the need for a red blood cell transfusion, this source of imprecision might remain a limitation.

The CBF calculation is strongly influenced by the reduced scattering coefficient, which is affected by the structure of the neonatal head. In particular, in a previous study performed by the same authors,^38^ it has been demonstrated that when homogeneous models are used to analyze TD-NIRS data, the reduced scattering coefficient is underestimated, and the error is larger in the case of preterm neonates with wider subarachnoidal space. However, by performing a Wilcoxon unsigned test (applying the Bonferroni correction), the CBF measured in previous studies performed on term neonates with the same device^39^^,^^40^ is not statistically significantly different from that reported in this study.

### 4.2 Physiological Interpretation

The increase in cerebral oxygenation would suggest a benefit of the transfusion in terms of increased oxygen availability and/or reduced risk of cerebral hypoxia if stressed by apnea or bradycardia with systemic hypoxemia, which is common in this population.

In the clinical situation of stable, growing preterm infants, however, it is surprising that correction of anemia results in increased cerebral oxygenation. The coupling between cerebral metabolism and cerebral blood flow, as well as the decreased blood viscosity in anemia, would both cause cerebral blood flow to increase with anemia to compensate for the reduced oxygen-carrying capacity and so, at least from a gross perspective, to maintain capillary oxygen tension—which is the driving force for the diffusion of oxygen toward the mitochondria of brain cells. Cerebral tissue oxygenation as measured by NIRS, however, is not capillary, and it is dominated by the hemoglobin in the blood in larger vessels, of which most is venous. Is it possible that transfusion induces a major shift in the proportion of arterial, capillary, and venous blood? If so, it would rather be in the direction of less arterial blood because it is the arteries that dilate in response to the decreased oxygen extraction due to the reduced oxygen-carrying capacity.

Another factor is the oxygen-hemoglobin dissociation curve, which may be left-shifted in bank blood due to GDP-6-phosphate depletion. This will indeed “require” a higher saturation to maintain the capillary oxygen tension.

The blood flow retrieved by DCS could be affected by Hct level; indeed, BFI is dependent on both the average flow and density of red blood cells in the interrogated tissue volume.^41^ In the case of RBCT, Hct level strongly increases after transfusion, biasing a before–after comparison. A correction factor between Hct level and BFI has been proposed.^42^ In a model, the Hct correction factor was used, c=1/((1-Hct·φ)), where φ was empirically determined to be 1.8. However, this factor was tested only on phantoms, and no *in vivo* validation has been reported. For the sake of completeness, we corrected the measured BFI and the corresponding ${\mathrm{tCMRO}}_{2}$ for the change in Hct as a result of the transfusion, obtaining BFI* and ${\mathrm{tCMRO}}_{2}$*. Both now increased, +31% for BFI* and +36% for ${\mathrm{tCMRO}}_{2}$*. These changes, however, are in the range of BFI repositioning imprecision (28%)^29^ and, more importantly, are very unexpected from physiological reasoning.

## 5 Conclusion

The TD NIRS technique confirmed that cerebral oxygenation is increased by red blood cell transfusion for anemia of prematurity. This increase was rather larger than what was detected by simultaneous SRS CW NIRS. Surprisingly, it was not possible to document the expected drop in cerebral blood flow, and disappointingly, the variation in the measure of cerebral metabolic rate of oxygen was too high for meaningful interpretation. Our perspective to draw clinical conclusions is to increase the number of observations and reduce the influence of tissue heterogeneity on CBF measured by DCS, thus the dispersion among different subjects.

## Data Availability

The data utilized in this study are available from the authors upon reasonable request.
